# Car Crashes and Central Disorders of Hypersomnolence: A French Study

**DOI:** 10.1371/journal.pone.0129386

**Published:** 2015-06-08

**Authors:** Fabio Pizza, Isabelle Jaussent, Regis Lopez, Carole Pesenti, Giuseppe Plazzi, Xavier Drouot, Smaranda Leu-Semenescu, Severine Beziat, Isabelle Arnulf, Yves Dauvilliers

**Affiliations:** 1 Sleep Disorders Center, Department of Neurology, Gui-de-Chauliac Hospital, CHU Montpellier, Montpellier, France; 2 Department of Biomedical and Neuromotor Sciences (DIBINEM), University of Bologna, Bologna, Italy; 3 IRCCS Istituto delle Scienze Neurologiche, ASL di Bologna, Bologna, Italy; 4 Inserm U1061, Montpellier, France, Université Montpellier 1, Montpellier, France; 5 National Reference Centre for Orphan Diseases, Narcolepsy, Idiopathic hypersomnia and Kleine-Levin Syndrome (CNR narcolepsie-hypersomnie), Paris, France; 6 CHU de Poitiers, Clinical Neurophysiology Department, 8600 Poitiers, France; 7 Sleep Disorders Unit, Pitié-Salpêtrière University Hospital, AP-HP, Brain Research Institute (CRICM-UPMC-Paris6, Inserm UMR_S 975, CNRS UMR 7225), Sorbonne Universities, UPMC Univ Paris 06, Paris, F-75005, France; Oasi research institute, ITALY

## Abstract

**Background:**

Drowsiness compromises driving ability by reducing alertness and attentiveness, and delayed reaction times. Sleep-related car crashes account for a considerable proportion of accident at the wheel. Narcolepsy type 1 (NT1), narcolepsy type 2 (NT2) and idiopathic hypersomnia (IH) are rare central disorders of hypersomnolence, the most severe causes of sleepiness thus being potential dangerous conditions for both personal and public safety with increasing scientific, social, and political attention. Our main objective was to assess the frequency of recent car crashes in a large cohort of patients affected with well-defined central disorders of hypersomnolence versus subjects from the general population.

**Methods:**

We performed a cross-sectional study in French reference centres for rare hypersomnia diseases and included 527 patients and 781 healthy subjects. All participants included needed to have a driving license, information available on potential accident events during the last 5 years, and on potential confounders; thus analyses were performed on 282 cases (71 IH, 82 NT2, 129 NT1) and 470 healthy subjects.

**Results:**

Patients reported more frequently than healthy subjects the occurrence of recent car crashes (in the previous five years), a risk that was confirmed in both treated and untreated subjects at study inclusion (Untreated, OR = 2.21 95%CI = [1.30–3.76], Treated OR = 2.04 95%CI = [1.26–3.30]), as well as in all disease categories, and was modulated by subjective sleepiness level (Epworth scale and naps). Conversely, the risk of car accidents of patients treated for at least 5 years was not different to healthy subjects (OR = 1.23 95%CI = [0.56–2.69]). Main risk factors were analogous in patients and healthy subjects.

**Conclusion:**

Patients affected with central disorders of hypersomnolence had increased risk of recent car crashes compared to subjects from the general population, a finding potentially reversed by long-term treatment.

## Introduction

Adequate alertness is a key pre-requisite to safely interact with the external environment and is physiologically modulated by sleep regulation in terms of homeostatic and circadian sleep propensity. Conversely, excessive daytime sleepiness (EDS, or hypersomnolence) refers to an individual inability to remain awake during daytime leading to multifaceted subjective feelings [1], executive functions deficits, as well as sleep occurrence in monotonous or even in inappropriate and potentially dangerous active situations [2]. The prevalence of EDS was recently reported by 27.8% of the general population thus representing a widespread symptom that may reflect inadequate lifestyles, including poor sleep hygiene, but also different underlying sleep disorders [3]. Narcolepsy type 1 (NT1, previously named narcolepsy with cataplexy), narcolepsy type 2 (NT2, previously named narcolepsy without cataplexy) and idiopathic hypersomnia (IH) are rare central disorders of hypersomnolence, the most severe causes of sleepiness thus being potential dangerous conditions for both personal and public safety with increasing scientific, social, and political attention [4,2]. Indeed, NT1 is characterized by EDS with sleep attacks and cataplexy (i.e. sudden loss of muscle tone evoked by strong emotions during wakefulness), both symptoms being linked to REM sleep dysregulation, and is pathophysiologically linked to the loss of hypocretinergic neurotransmission as detectable at the cerebrospinal level [5]. Compared to NT1, NT2 is characterized by EDS with sleep attacks and neurophysiological evidence of occurrence of REM sleep at sleep onset (sleep onset REM periods, SOREMPs), whereas in IH the EDS manifests without clinical or neurophysiological evidence of REM sleep dysregulation. Moreover, both NT2 and IH patients typically show normal cerebrospinal hypocretin-1 levels, thus being hypocretin deficiency an exclusive biomarker of NT1 (also viewed as the hypocretin deficiency syndrome) [2].

Over the last two decades, sleepiness has shown a strong causal role in the multifactorial determination of road accidents [6, 7]. Indeed, while sleepiness seems the primary cause of 1 to 3% of car crashes [8], it influences up to 20% of them [6,9], especially those occurring during nighttime or in monotonous driving conditions such as highways, and frequently involving young male drivers [8]. In this context, self-reported near miss accidents appeared a dose response predictor of actual car crash history in the general population and were associated with high subjective sleepiness complaint on the Epworth sleepiness scale (ESS), being the most widely used questionnaire to quantify the severity of daytime sleepiness [10,11]. In addition, the daytime sleep propensity may also be measured objectively by the Multiple Sleep Latency Test (MSLT), a standardized neurophysiological measurement that consists of five nap opportunities scheduled at two-hour intervals with instruction given to try to fall asleep [12]. Hence, MSLT sleep latency has been shown to predict the subsequent occurrence of verified car crashes documented at 10 year follow-up in the general population [13].

Among sleep disorders with prominent EDS symptomatology, obstructive sleep apnea syndrome (OSAS) has been widely investigated in terms of driving risk [14,15]; however scarce data are available in patients with central disorders of hypersomnolence [16]. One study showed that patients with clinically-defined narcolepsy/cataplexy reported more driving accidents than controls and patients with epilepsy, but did not consider the impact of treatment [17]. Aldrich also assessed the occurrence of crashes and accidents due to sleepiness in different sleep disorders at diagnostic evaluation compared to controls, and found a higher proportion of individuals with sleep-related accidents in the hypersomnia groups (narcolepsy and other sleep disorders with EDS) than in the control group [18]. Recent (i.e. within 5 years) driving accidents or near misses were reported by 55% of drug-naive hypersomnia patients (75% of NT1, 50% of NT2 and IH) and associated with high ESS scores in an uncontrolled Japanese study [19]. Two large internet-based surveys focused on the relationship between near-miss accidents and crash risk showing an additional association between self-reported sleep disorder diagnoses (OSAS, narcolepsy, hypersomnia or multiple sleep disorders) and accidents occurrence [11,20]. Thus, the available data with controlled central hypersomnia diagnoses are old, rare, and suffer from methodological limitations on outcome assessment and potential confounders [17,18], while larger studies neither investigate disease characteristics and related-medication intake nor objectively confirm self-reported diagnoses [11,20]. Indeed, European Countries have different Driving License regulations and struggle to reach a common policy in primis for patients with OSAS [21]. Therefore controlled data on a large cohort of patients affected with well-defined central hypersomnias, whether treated or drug-naive, compared to drivers from the general population are highly warranted to adequately treat and support these at-risk populations. The aim of this cross-sectional study was to assess the driving accident risk in the largest reported population of patients with definite diagnoses of different central disorders of hypersomnolence versus healthy subjects taking into account main features influencing accidental risk as in the epidemiological studies, as well as disease-related characteristics and the impact of short vs long-term medication.

## Methods

### Participants

Overall, 527 adult patients (median age: 36.2 years (range = 18.0–85.9); 57.7% women) with a central disorder of hypersomnolence were recruited and classified based on their primary ICSD-3 diagnosis [2]: NT1 (n = 291), NT2 (n = 132), and IH (n = 104). Data were collected from a national research program (NARCOBANK study) on narcolepsy and other central disorders of hypersomnolence performed in French reference centres for rare hypersomnia diseases between 2008 and 2011.

Patients were selected on the basis of the following criteria to fulfill current diagnostic requirements [2]: 1) age ≥ 18 years old; 2) definite diagnosis of NT1 (i.e. history of definite cataplexy and mean MSLT sleep latency ≤ 8 minutes with ≥ 2 SOREMPs or cerebrospinal hypocretin-1 deficiency), of NT2 (i.e. mean MSLT sleep latency ≤ 8 minutes with ≥ 2 SOREMPs, no cataplexy and normal cerebrospinal hypocretin-1 levels if performed), and IH (i.e. normal polysomnography, mean MSLT sleep latency < 8 minutes with < 2 SOREMPs and normal CSF hypocretin-1 levels if performed). IH defined as total sleep time greater than 11h/24h on long-term polysomnography with MSLT latencies greater than 8 min was not included in the present study [2]. The presence and dose of psychostimulant medication, disease and treatment duration, occurrence of irresistible sleep attacks, frequency of partial and global cataplectic attacks (in NT1 patients only) and the ESS score at time of study were reported for each patient [10]. Untreated patients were drug naïve subjects included at diagnostic assessment.

Adult healthy subjects (n = 781, median age: 28.29 years (range = 18.04–81.20); 68.3% women) were recruited via advertisement during the same period from the general population according to the following criteria: 1) age≥ 18 years old; 2) males or females; 3) ability to currently speak and comprehend French; 4) consent to participate to the study; 5) absence of significant medical, neurological and psychiatric diseases.

All participants agreed to take part in this research program that includes a systematic interview, a clinical examination, completion of questionnaires, and a biological collection. All patients and healthy subjects gave their written informed consent for the study that was approved by the local ethics committees (Comité de Protection des Personnes–Ile de France 06).

### Main study outcome: Car crashes

Car crashes as a driver during the past five years were recorded at study inclusion by means of a self-administered questionnaire. Patients and healthy subjects with a regular driving license were selected from the database, and then dichotomized considering the response to “Have you had a car accident in the previous five years?”

### Demographic, clinical and neurophysiological variables

For all patients and healthy subjects the standardized questionnaire included questions on 1) gender; 2) age at inclusion; 3) educational level (dichotomized as high (≥12 years) and low (<12 years)); 4) civil status (considered as unmarried versus all the other conditions including married/couple, separated/divorced, and widower); 5) body mass index (BMI) category (<25, 25–30, >30 kg/m^2^); 6) consumption of coffee, tea, energy drinks, and alcohol; 7) smoking; 8) ESS score at study inclusion that measures the probability of dozing-off in eight different situations leading to a final score from 0 to 24 being pathological above 11 [10]; 9) habit to perform a regular daily nap; and 10) self-reported driving exposure (kilometers driven per year). For sub-analyses the following additional disease-related features were considered in patients only: 1) treatment status, duration, and type at the inclusion; 2) diagnosis category; 3) disease duration; 4) mean sleep latency at the MSLT at diagnosis; 5) apnea-hypopnea index at diagnosis; and 6) occurrence of irresistible sleep attacks and (in NT1 patients only) of cataplectic attacks.

### Statistical analysis

Cases and healthy subjects were compared using logistic regression models. Associations between subject characteristics, treatment and diagnosis category were quantified with odds ratios (OR) and their 95% confidence intervals (CI). Age, gender, socio-demographic, and clinical and neurophysiological variables associated with car accidents in univariate analyses (with p<0.15) were included in logistic regression models to estimate adjusted OR for the relationships between car crash occurrence and 1) treatment group (healthy subjects, drug-naïve cases without treatment, and cases with treatment); 2) treatment exposure (healthy subjects, drug-naïve cases without treatment, cases with treatment for < 5 years, and cases with treatment for ≥ 5 years); and 3) diagnostic category. When appropriate, the interaction terms were tested using the Wald-χ^2^ test given by the logistic regression model. Significance level was set at p<0.05. Analyses were performed using SAS statistical software (version 9.3; SAS Inc, Cary, North Carolina).

## Results

### Subject profiles

**Fig 1:**
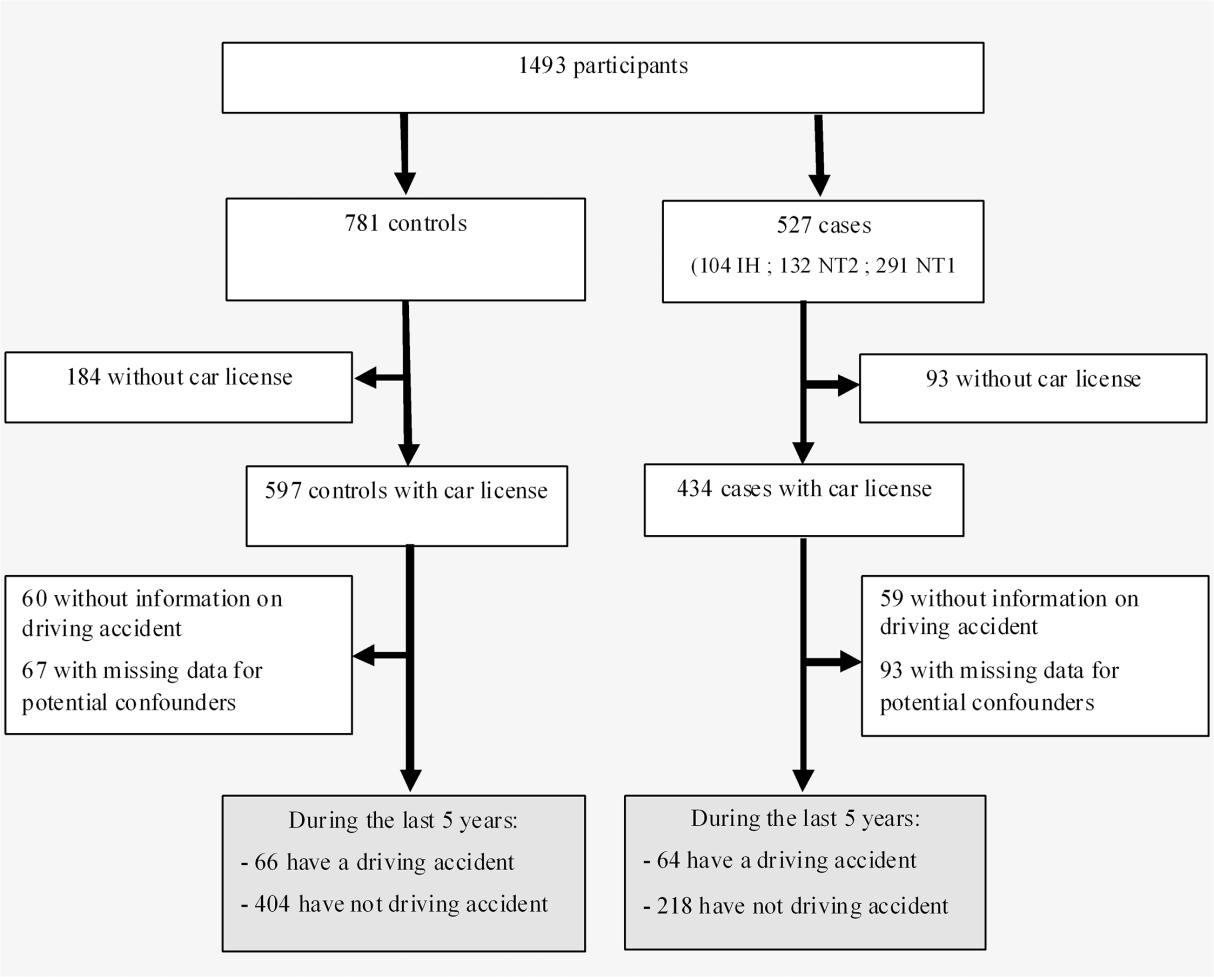
Flow chart of patients and healthy subjects.

All subjects included needed to have a valid driving license, information available on potential accident events during the last 5 years, and on potential confounders (i.e. gender, age, educational level, BMI, coffee, tea, energy drinks, alcohol consumptions, smoking status, ESS score, and medication intake if any for patients). The present analyses were thus performed on a population of 470 healthy subjects and 282 cases (71 IH, 82 NT2, 129 NT1) (Fig 1). Among the 282 patients, 173 (61.35%) took psychostimulants at study inclusion. Among them, 103 (59.54%) took modafinil, 42 (24.28%) methylphenidate, 17 (9.83%) sodium oxybate, 6 (3.47%) mazindol, with median [range] doses respectively of 400 (100–600) mg/day, 36 (5–112) mg/day, 6.75 (2.15–9.00) g/day, and 4 (1–4) mg/day, and only 5 patients took others stimulants such as pitolisant or amphetamines.

Compared to cases included, cases excluded from the study were significantly more likely to be older and more overweight or obese (p<0.05, for all comparisons), without significant differences for car crashes occurrence, substance use, and ESS. In addition, no significant differences were found between healthy subjects excluded and those included on gender, age, educational level, marital status, BMI, coffee, tea, energy drinks and alcohol consumptions, smoking status and car crashes. Compared to healthy subjects, patients finally included were significantly more likely to be older, male, had a lower education level, were more overweight/obese, smoker, taking more coffee, and alcohol, but less tea, higher self-reported driving exposure, and as expected had higher daytime sleepiness assessed by naps and ESS scores (Table 1, p≤0.01 for all the comparisons). We also found significant differences between groups for driving accident during the last 5 years with 14% (n = 66) in healthy subjects and 22.7% (n = 64) in patients (p = 0.003). Results remained unchanged after adjustment for socio-demographic (sex, age, BMI, education level), health and behaviour status (smoking status, coffee, alcohol and tea consumptions) and driving exposure associated at p<0.10 in Table 1 (p = 0.01).

**Table 1:** Socio-demographic, health behaviour and health status factors according to healthy subjects and cases.

	*Healthy subjectsN = 470*		*CasesN = 282*		
*Variable*	*n*	*%*	*n*	*%*	*p*
Women ^(1)^	324	68.94	156	55.32	0.0002
Age at inclusion (in years) ^(2)^	31 [18–79]		37 [18–83]		0.0006
Age at inclusion (in years) ≥65^(3)^	26	5.53	22	7.80	0.22
High educational level ^(4)^	389	82.77	206	73.05	0.002
Unmarried ^(5)^	164	34.89	88	31.21	0.30
BMI (kg/m^2^)					
<25	351	74.68	141	50.00	<0.0001
25–30	88	18.72	93	32.98	
>30	31	6.60	48	17.02	
Coffee intake ^(6)^	298	63.40	207	73.40	0.005
Tea intake ^(6)^	222	47.23	104	36.88	0.006
Energy drinks ^(6)^	157	33.40	97	34.40	0.78
Alcohol intake ^(6)^	242	51.49	171	60.64	0.01
Current smoker ^(6)^	99	21.06	92	32.62	0.0005
ESS >10 ^(7)^	20	4.26	250	88.65	<0.0001
Nap ^(6)^	193	41.06	233	85.04	<0.0001
Kilometers driven per year					
<2000	114	28.36	41	18.22	0.004
2000–6000	114	28.36	54	24.00	
6001–13000	87	21.64	62	27.56	
>13000	87	21.64	68	30.22	
Driving accident during the last 5 years ^(6)^	66	14.04	64	22.70	0.003

^(1)^ Women vs Men

^(2)^ Continuous variables were expressed by Median [Minimum value-Maximum value]

^(3)^ ≥65 vs <65

^(4)^ High vs Low

^(5)^ Unmarried vs Married, Couple, Separated, Divorced, Widower.

^(6)^ Yes vs No

^(7)^ >10 vs ≤ 10

### Driving accidents in the previous 5 years: association and impact of central hypersomnias

Comparison between subjects with (n = 130) and without (n = 622) history of recent driving accidents revealed that subjects from the former group were more likely male, younger, unmarried, user of energy drinks, and with higher levels of subjective sleepiness at the ESS (p ≤ 0.01, for all comparisons) with tendencies for less coffee consumption and having a regular daily nap (p<0.10, for these comparisons) (Table 2). Subsequent analyses were thus adjusted for these factors.

**Table 2:** Socio-demographic, health behaviour and health status factors according to driving accident during the last 5 years.

	*Driving accident during the last 5 years*					
	*NoN = 622*		*YesN = 130*			
*Variable*	*n*	*%*	*n*	*%*	*OR [95% CI]*	*p*
Women ^(1)^	410	65.92	70	53.85	0.60 [0.41;0.88]	0.01
Age at inclusion (in years) ^(2)^	36.79 [18.45–83.54]		27.77 [18.75–73.91]		0.96 [0.95;0.98]	<0.0001
Age at inclusion (in years) ≥65^(3)^	45	7.23	3	2.31	0.30 [0.09;0.99]	0.05
High educational level ^(4)^	487	78.30	108	83.08	1.36 [0.83;2.24]	0.22
Unmarried ^(5)^	196	31.51	56	43.08	1.64 [1.12;2.42]	0.01
BMI (kg/m^2^)						
<25	408	65.59	84	64.62	1	0.20
25–30	154	24.76	27	20.77	0.85 [0.53;1.36]	
>30	60	9.65	19	14.62	1.54 [0.87;2.71]	
Coffee intake ^(6)^	427	68.65	78	60.00	0.68 [0.46;1.01]	0.06
Tea intake ^(6)^	273	43.89	53	40.77	0.88 [0.60;1.29]	0.51
Energy drinks ^(6)^	189	30.39	65	50.00	2.29 [1.56;3.36]	<0.0001
Alcohol intake ^(6)^	338	54.34	75	57.69	1.15 [0.78;1.68]	0.49
Current smoker ^(6)^	157	25.24	34	26.15	1.05 [0.68;1.61]	0.83
ESS >10 ^(7)^	211	33.92	59	45.38	1.62 [1.10;2.37]	0.01
Nap ^(6)^	343	55.68	83	64.84	1.47 [0.99;2.18]	0.06
Kilometers driven per year						
<2000	126	24.37	29	26.36	1	0.86
2000–6000	139	26.89	29	26.36	0.91 [0.51;1.60]	
6001–13000	126	24.37	23	20.91	0.79 [0.44;1.45]	
>13000	126	24.37	29	26.36	1.00 [0.57;1.77]	

^(1)^ Women vs Men

^(2)^ Continuous variables were expressed by Median [Minimum value-Maximum value]

^(3)^ ≥65 vs <65

^(4)^ High vs Low

^(5)^ Unmarried vs Married, Couple, Separated, Divorced, Widower.

^(6)^ Yes vs No

^(7)^ >10 vs ≤ 10

The comparisons between patients with central hypersomnia and healthy subjects for the risk of recent car accidents are shown in Table 3. The risk to have a driving accident was significantly higher in cases whether treated or drug-naïve at inclusion in crude association, results remaining significant after adjustment (Model 1, p = 0.002). When adjusting also for ESS and regular daily napping, being intrinsic markers of the hypersomnia diseases, the associations were borderline significant (Model 2; p = 0.07). Further dissecting the clinical population for treatment exposure showed that patients treated for more than 5 years were not at increased risk compared to healthy subjects in both crude associations and adjusted models (Model 1: drug-naïve condition, OR = 2.20 95%CI = [1.29–3.73], treated for ≤ 5 years condition OR = 2.68 95%CI = [1.56–4.62], treated for > 5 years condition OR = 1.23 95%CI = [0.56–2.69], respectively). An association was also found between the category of central hypersomnia and driving accident, with higher risk for NT2 and IH, and with similar tendency for NT1 in comparison to healthy subjects (OR = 1.68 95%CI = 0.97–2.91 for NT1; OR = 2.82 95%CI = [1.60–4.96] for NT2; and OR = 2.04 95%CI = [1.05–3.95] for IH, p = 0.002). No significant interaction was found between category of diagnosis, treatment and car accident (p = 0.92).

**Table 3:** Between-group (patients and healthy subjects) comparisons for the driving accident during the last 5 years.

	*Driving accident during thelast 5 years*									
	*NoN = 622*		*YesN = 130*		*Model 0*		*Model 1*		*Model 2*	
*Variable*	*n*	*%*	*n*	*%*	*OR [95% CI]*	*p*	*OR [95% CI]*	*p*	*OR [95% CI]*	*p*
Conditions										
Healthy subjects	404	64.95	66	50.77	1	0.006	1	0.002	1	0.07
Patients without treatment	81	13.02	28	21.54	2.12 [1.28;3.50]		2.21 [1.30;3.76]		2.62 [1.10;6.26]	
Patients with treatment	137	22.03	36	27.69	1.61 [1.03;2.52]		2.04 [1.26;3.30]		2.39 [1.06;5.36]	
Category of subjects and drug exposure										
Healthy subjects	404	65.37	66	50.77	1	0.0009	1	0.0007	1	0.04
Patients without treatment	81	13.11	28	21.54	2.12 [1.28;3.50]		2.20 [1.29;3.73]		2.43 [1.02;5.81]	
Patients with treatment for ≤ 5 years	71	11.49	27	20.77	2.33 [1.39;3.89]		2.68 [1.56;4.62]		2.90 [1.27;6.62]	
Patients with treatment for > 5 years	62	10.03	9	6.92	0.89 [0.42;1.87]		1.23 [0.56;2.69]		1.41 [0.49;4.06]	
Category of central disorders of hypersomnolence										
None	404	64.95	66	50.77	1	0.006	1	0.002	1	0.06
NT1	104	16.72	25	19.23	1.47 [0.89;2.45]		1.68 [0.97;2.91]		1.90 [0.77;4.65]	
NT2	58	9.32	24	18.46	2.53 [1.47;4.36]		2.82 [1.60;4.96]		3.14 [1.32;7.44]	
IH	56	9.00	15	11.54	1.64 [0.88;3.07]		2.04 [1.05;3.95]		2.31 [0.94;5.71]	

Model 0: Crude association; Model 1: Adjustment for gender, age, unmarried status, coffee intake, and energy drinks consumption; Model 2: Adjustment for all the covariates in model 1 plus ESS and naps.

### Risk factors for car crashes in patients with central hypersomnia

Subsequent analyses were performed in patients exclusively to disentangle potential intrinsic risk factors for car accidents. The risk of car accidents increased significantly in subjects being male, younger, unmarried, using frequently energy drinks, and having shorter disease duration. No other significant associations were found between clinical (i.e. mainly BMI, ESS, irresistible sleep attacks and naps), neurophysiological features (MSLT latency) and driving accidents (Table 4). None of patients with a car crash presented an apnea-hypopnea index above 15 per hour at time of diagnosis and only 10.7% in the group without any accident (Table 4). Being medicated by a psychostimulant at study inclusion, using different types of treatments (drug free, modafinil, methylphenidate, and others), and having a specific diagnosis category did not show any significant association with driving accident neither in crude nor in adjusted models (Table 5). However, the comparison among groups with different duration of treatment exposure (below and above 5 years) was borderline significant in crude associations (p = 0.06) confirming the potential protective role of long term stable treatment (Model 1: treated for ≤ 5 years condition OR = 1.10 95%CI = [0.59–2.04], treated for > 5 years condition OR = 0.42 95%CI = [0.18–0.95], respectively). However this association failed to be significant after adjusting for gender, age, civil status, energy drinks consumption, and disease duration.

**Table 4:** Socio-demographic, health behavior, health status and sleep factors according to driving accident during the last 5 years in patients with central disorders of hypersomnolence.

	*Driving accident during the last 5 years*					
	*NoN = 218*		*YesN = 64*			
*Variable*	*n*	*%*	*n*	*%*	*OR [95% CI]*	*p*
Women ^(1)^	130	59.63	26	40.63	0.46 [0.26;0.82]	0.008
Age at inclusion (in years) ^(2)^	39.34 [18.45–83.54]		30.38 [18.75–73.91]		0.96 [0.94;0.98]	0.0008
Age at inclusion (in years) ≥ 65 ^(3)^	19	8.72	3	4.69	0.52 [0.15;1.80]	0.30
High educational level ^(4)^	156	71.56	50	78.13	1.42 [0.73;2.75]	0.30
Unmarried ^(5)^	60	27.52	28	43.75	2.05 [1.15;3.64]	0.01
BMI (kg/m^2^)						
<25	104	47.71	37	57.81	1	0.28
25–30	77	35.32	16	25.00	0.58 [0.30;1.13]	
>30	37	16.97	11	17.19	0.84 [0.39;1.81]	
Coffee intake ^(6)^	163	74.77	44	68.75	0.74 [0.40;1.37]	0.34
Tea intake ^(6)^	83	38.07	21	32.81	0.79 [0.44;1.43]	0.44
Energy drinks ^(6)^	66	30.28	31	48.44	2.16 [1.22;3.82]	0.008
Alcohol intake ^(6)^	131	60.09	40	62.50	1.11 [0.62;1.97]	0.73
Current smoker ^(6)^	74	33.94	18	28.13	0.76 [0.41;1.41]	0.38
Kilometers driven per year						
<2000	34	19.65	7	13.46	1	0.73
2000–6000	41	23.70	13	25.00	1.54 [0.55;4.29]	
6001–13000	48	27.75	14	26.92	1.42 [0.52;3.88]	
>13000	50	28.90	18	34.62	1.75 [0.66;4.64]	
Disease duration, >5 years ^(7)^	129	59.17	28	43.75	0.54 [0.31;0.94]	0.03
MSLT (in min) ^(2)^	5.00 [0.40–17.00]		4.90 [0.40–12.60]		0.93 [0.84;1.04]	0.23
ESS^(2)^	17.00 [4.00–24.00]		17.00 [5.00–24.00]		1.02 [0.96;1.09]	0.50
ESS						
<11	24	11.01	8	12.50	1	0.55
11–16	81	37.16	19	29.69	0.70 [0.27;1.81]	
>16	113	51.83	37	57.81	0.98 [0.41;2.37]	
Irresistible sleep attacks ^(6)^	180	83.72	54	88.52	1.50 [0.63;3.57]	0.36
Nap ^(6)^	184	86.79	49	79.03	0.57 [0.28;1.19]	0.14
AHI						
≤5	131	66.84	48	80.00		NA
]5–15]	44	22.45	12	20.00		
]15–30]	12	6.12	0	0.00		
>30	9	4.59	0	0.00		

^(1)^ Women vs Men

^(2)^ Continuous variables were expressed by Median [Minimum value-Maximum value]

^(3)^ ≥65 years vs <65 years

^(4)^ High vs Low

^(5)^ Unmarried vs Married, Couple, Separated, Divorced, Widower.

^(6)^ Yes vs No

^(7)^ >5 years vs ≤5 years

NA: Not applicable

**Table 5:** Between-group comparisons for the driving accident during the last 5 years among patients with central disorders of hypersomnolence.

	*Driving accident during thelast 5 years*							
	*NoN = 218*		*YesN = 64*		*Model 0*		*Model 1*	
*Variable*	*n*	*%*	*n*	*%*	*OR [95% CI]*	*p*	*OR [95% CI]*	*p*
Medication								
Drug-free treatment	81	37.16	28	43.75	1	0.34	1	0.70
Psychostimulant intake	137	62.84	36	56.25	0.76 [0.43;1.34]		0.88 [0.48;1.64]	
Medication exposure								
Drug-free treatment	81	37.85	28	43.75	1	0.06	1	0.28
Use of psychostimulant for less than 5 years	71	33.18	27	42.19	1.10 [0.59;2.04]		1.30 [0.60;2.79]	
Use of psychostimulant for more than 5 years	62	28.97	9	14.06	0.42 [0.18;0.95]		0.55 [0.22;1.34]	
Type of medication								
Drug-free treatment	81	38.21	28	44.44	1	0.78	1	0.98
Modafinil	78	36.79	22	34.92	0.82 [0.43;1.55]		0.92 [0.46;1.84]	
Methylphenidate	33	15.57	9	14.29	0.79 [0.34;1.85]		0.92 [0.36;2.33]	
Others	20	9.43	4	6.35	0.58 [0.18;1.84]		0.78 [0.23;2.66]	
Diagnostic category								
NT1	104	47.71	25	39.06	1	0.24	1	0.26
NT2	58	26.61	24	37.50	1.72 [0.90;3.28]		1.81 [0.89;3.68]	
IH	56	25.69	15	23.44	1.11 [0.54;2.28]		1.37 [0.63;3.00]	

^(1)^Continuous variables are expressed by Median [Minimum value-Maximum value]

Model 0: crude association; Model 1: Adjustment for gender, age, civil status, energy drinks consumption, and disease duration.

Sensitivity analyses were also performed 1/ after excluding 21 patients and 106 healthy subjects with age < 23 years (to fully collect potential reported accident during the last five years as minimal age of inclusion was 18 y.o.), 2/ after excluding healthy subjects with ESS scores above 10 (n = 20), and 3/ after excluding healthy subjects and patients above 65 years old, and all results remained unchanged (data not shown).

## Discussion

We explored the proportion of self-reported accidents at the wheel in the last five years in the largest reported population of well-defined central disorders of hypersomnolence versus driversfrom the general population. Patients reported more frequently than healthy subjects the occurrence of recent car crashes, a risk that was confirmed in both treated and drug-naïve subjects at the time of inclusion, as well as in all disease categories, and was modulated by subjective sleepiness level (ESS and daily naps, intrinsic severity measures). Conversely, the risk of car accidents of patients treated for at least 5 years was comparable to that of the general population.

The finding of increased driving accident risk in central disorders of hypersomnolence is in line with the scientific evidences on the role of sleepiness in the multifactiorial determination of crash risk [6,11,13,20], and further extends previous data suggesting an increased risk in patients with narcolepsy [17,18], and with other (non-apneic) sleep disorders associated with EDS [18]. Indeed, our findings showed for the first time that the increased crash risk pertained also to patients with NT2 and IH, while patients with IH with prolonged sleep at 24 hour monitoring and normal MSLT latency were excluded to fulfil similar inclusion criteria on daytime sleep propensity as for NT1 and NT2. While previous clinical-based studies showed increased risk of accidents subjectively attributed to sleepiness [17,18], our data analysis disclosed an overall increased occurrence of recent crashes with EDS (i.e. high ESS score and daily napping) being among the intrinsically modulating factors, as recently suggested [19]. Therefore the present finding bridges old data to recent epidemiological studies where self-reported diagnoses of “narcolepsy” [11], or of “narcolepsy and hypersomnia” [20], were associated with higher occurrence of both near misses and accidents. Moreover, the accidental risk we found is comparable to previous data in OSAS disclosing a 2 to 3 times increased risk for untreated patients as well as a positive impact of the adequate ventilatory treatment in decreasing the risk [14,15,22]. The increased risk of car crashes we found in central disorders of hypersomnolence was however not related to comorbid OSAS. We also reported in the current study that the risk of car accidents increased significantly in subjects using frequently energy drinks. Although being potentially a counter-intuitive result, recent studies also reported that subjects who consumed energy drinks were more likely to be daytime sleepers [23].

While large studies on car accidents with controlled series of central hypersomnia are scarce, several authors addressed objective driving performance with computer based simulators of different complexity [24–26], up to high tech approaches integrated into a real car simulator [27]. Simulated driving performance includes several objective measures that are used as surrogate markers of real driving ability in monotonous conditions, encompassing primary vehicle control (i.e. tracking performance, accidents) and other secondary tasks testing individual ability to interact with the environment while driving (e.g. reaction times), that showed good external validity compared to real driving [28]. Driving simulator studies were consistent in showing a poor driving performance in mixed populations of narcoleptic patients with and without cataplexy [24–26], or of patients with a mixture of central disorders of hypersomnolence [27]. Laboratory-based studies also confirmed the intrinsic relations in central disorders of hypersomnolence between objective measures of driving performance and sleepiness assessed by either MSLT [24,25], or maintenance wakefulness test (MWT) [27]. The MWT is to date the best objective driving performance predictor among different sleepiness measures in OSAS patients [29,30], and the relation between objective driving performance and accident rate appeared also modulated by behavioural attitudes (e.g. stopping driving) towards driving while perceiving sleepiness [31]. Accordingly, a previous case-control study focusing on driving behaviour and crash history disclosed that narcoleptic patients over 40 years usually adopted efficacious coping strategies (napping or taking medications before driving, stopping over to take a nap while feeling sleepy at the wheel) resulting in even less crashes than controls [32]. In addition, modafinil significantly improved real driving ability in patients with narcolepsy and IH in parallel with alertness levels at the MWT [33]. Indeed, we found that the stable long-term use of psychostimulants (at least for 5 years, the time span of accidents assessment) protected patients from the driving risk. Conversely, while sleepiness measures (baseline MSLT latency or ESS score at time of study) were not directly associated with driving accidents, subjective sleepiness appeared as a modulating key factor at multivariate analyses.

We emphasize the need to implement current guidelines for driving license legislations across Europe taking into account the risk of sleepiness at the wheel in the general population, OSAS, and in central disorders of hypersomnolence, as well as to reach a common social support policy in order to reduce the burden of sleepiness and to better integrate sleepy patients at social level [7,21]. Indeed, it has been strongly recommended to treat OSAS with continuous positive airway pressure, to inquire on additional potential causes of sleepiness, to investigate driving risk and to educate patients on the risks of drowsy driving [34]. In addition, recent Canadian guidelines also proposed to include sleep physician evaluation of symptoms, adequate treatment and compliance for certifying fitness to drive in OSAS patients [35]. While other chronic diseases share easily measurable disability profiles, central disorders of hypersomnolence suffer from an “invisible” form of disability for the intrinsic nature of EDS and need chronic treatment, education, and assistance. Indeed, when patients are well-informed about their disease and routinely apply adequate coping strategies along disease course their driving risk may be similar to the general population [32]. Indeed, long-term treated patients showed a risk profile comparable to healthy subjects from the general population and probably reflected the concurrent achievement of both adequate symptoms management and increased disease awareness. This finding may explain also the overall lower disease-related burden of patients who received an early diagnosis [36]. Accordingly, new guidelines for the examination of patients with narcolepsy and related central hypersomnia for driving license should include the presence and severity of symptoms, their effects on daily function, compliance to treatment and efficacy of medications, and the use of adequate coping strategies together with objective alertness tests (MWT) [37–39]. There is a real need for systematic and standardized outcome assessment instruments specific for narcolepsy and related-hypersomnia to facilitate effective long-term management and to guarantee the right to drive to patients especially for professional drivers (e.g. truck drivers, taxi drivers, etc) without exposing them and the society to any accidental risk [39]. Recent studies also suggested an increased mortality of not yet known origin in patients with narcolepsy but possibly negatively influenced by accidents [40,41]. Indeed, appropriate legislations, together with early diagnosis, and cultural/social interventions to better integrate these patients into their society will hopefully improve the quality of life of patients and their families [42].

This study also suffered from limitations. First, accidents were assessed by means of self-reported data for the past five years only. The self-reported approach precludes any inference on fatal accidents occurrence, and the questionnaire did not include any item on timing, road type, consequence or responsibility (i.e. actively vs passively involved) of the crash. Second, the cross sectional study design forbids any inference on accidental risk reduction after treatment at individual level. Third, neither objective measures of alertness (i.e. MWT), near-miss accidents evaluation nor information on routinely adopted coping strategies towards drowsy driving were available to further disentangle the relation between treatment efficacy, individual behaviour and accidental risk. Fourth, albeit healthy subjects did not undergo polysomnographic studies and may thus have undiagnosed sleep disorders (including OSAS) as in the general population, we were able to find significant associations on accidental outcomes.

## Conclusion

Patients with central disorders of hypersomnolence as a whole have an increased risk of recent accidents at wheel that is potentially reversed by stable treatment. Further longitudinal studies are needed to evaluate the individual effect of treatment, the behavioural attitudes together with objective alertness levels on individual driving risk across the course of different categories of hypersomnia diseases.
